# Theoretical Calculations on Hexagonal-Boron-Nitride-(h-BN)-Supported Single-Atom Cu for the Reduction of Nitrate to Ammonia

**DOI:** 10.3390/molecules30244700

**Published:** 2025-12-08

**Authors:** Guoliang Liu, Cen Hao

**Affiliations:** School of Information Technology, Jiangsu Open University, Nanjing 210017, China

**Keywords:** theoretical calculations, h-BN, single-atom catalysis (SAC), nitrate reduction reaction (NO_3_RR)

## Abstract

Nitrate (NO_3_^−^), as a stable nitrogen-containing compound, has caused serious harm to the ecological environment and human health. To reduce nitrate pollution, the catalytic reduction of nitrate (NO_3_RR) to ammonia (NH_3_) is a very promising solution. Recently, single-atom catalysts (SACs) have received extensive attention due to their excellent activity and stability. Here, we study the nitrate catalytic reduction properties of hexagonal-boron-nitride-(h-BN)-supported single-atom Cu systematically and theoretically and compare it with monolayer h-BN. We find that (1) due to the stronger electronegativity of the N atom, Cu atom is preferentially doped at the N top site, resulting in the significant electron rearrangement; (2) the doped Cu atom at the N top site for monolayer h-BN can provide extra 3d-orbital electrons at the Fermi level, which can significantly enhance the conductivity, reduce the bandgap width, and increase the reducibility; (3) the NO_3_^−^ ion preferentially adsorbs at the hollow site of monolayer h-BN, while the NO_3_^−^ ion is adsorbed more strongly at the Cu top site of h-BN-supported single-atom Cu due to the abundant d-electron supply from the Cu atom; (4) single-atom Cu can significantly reduce the energy barrier of the rate-determining step (RDS) and increase the probability of nitrate reduction. In conclusion, h-BN-supported single-atom Cu exhibits excellent catalytic performance of NO_3_RR.

## 1. Introduction

Due to the disruption of the natural nitrogen cycle by humans, as the most stable form of nitrogen-containing compound in an oxygen-rich environment, nitrate (NO_3_^−^) is widely present in groundwater, rivers, lakes, and coastal water [1,2,3]. Excessive NO_3_^−^ can lead to water eutrophication, seriously damaging the water ecosystem and further causing air pollution [4,5]. For human health, NO_3_^−^ in drinking water can increase the risk of people falling ill [6,7]. To address this issue, the reduction of NO_3_^−^ (NO_3_RR) to NH_3_ offers a very promising solution. This method not only removes harmful NO_3_^−^, but also can synthesize NH_3_, a prominent chemical that is commonly used as a raw material in industry [8,9].

The reduction reaction of the NO_3_^−^ ion (NO_3_RR) converted to NH_3_ by catalysis involves nitrogen-contained species with different valence states from +5 to −3 and is a complicated eight-electron process [10]. The reaction is given by the following equation:

(1) $${\mathrm{NO}}_{3}^{-}{+9H}^{+}{+8e}^{-}\to {\mathrm{NH}}_{3}{+3H}_{2}O$$

It can be seen from the above equation that the NO_3_RR is a complex process involving multi-electron coupled proton transfer, and it faces many challenges, which limits its practical application. Firstly, the reaction needs to overcome multiple kinetic energy barriers, resulting in an excessively high overpotential and sluggish rates [11]. Secondly, the presence of the competing hydrogen evolution reaction (HER) leads to a decrease in reaction efficiency [12]. Furthermore, during the reaction process, various intermediate products (NO_2_^−^, NO, N_2_O, N_2_, NH_2_OH, etc.) will be generated, which reduces the selectivity of NH_3_ production [13,14]. Therefore, reducing the overpotential, enhancing NH_3_ selectivity, and inhibiting HER and the formation of intermediates are of great significance for efficiently achieving the conversion from NO_3_^−^ to NH_3_.

Single-atom catalysts (SACs) exhibit high potential for efficient NH_3_ synthesis during the reduction reaction of the NO_3_^−^ ion (NO_3_RR) due to the maximized atomic utilization and distinctive electronic structure [15,16,17]. SACs used for NO_3_RR mainly include the following categories: Cu-SACs, Fe-SCAs, Zn-SACs, Co-SACs, and single-atom alloys [18,19,20,21]. Among them, due to the abundant unpaired d-electrons, Cu-based catalysts have a tunable electronic structure, high abundance, flexible electrochemical activity, and are regarded as one of the optimal candidates for NO_3_RR [22,23]. For instance, Chunhua Feng et al. [24] have proved single-atom Cu catalysts can enhance the rate of nitrate conversion to ammonia because of the strong binding between Cu and N; Xiaoqin wang et al. [25] have experimentally demonstrated boron nitride-supported copper single-atom catalyst exhibits significantly high NH_3_ productivity in the NO_3_RR.

As an analog of graphene, hexagonal boron nitride (h-BN) is considered a new promising catalytic material due to its high chemical stability, low cost, and its low environmental impact. At present, h-BN has already been used as a catalyst in reactions such as CO_2_ reduction [26], N_2_ fixation [27], and others [28]. However, the wide bandgap energy limits the catalytic applications of h-BN. There have been a series of studies that attempted to enhance the catalytic properties of h-BN by doping metallic or non-metallic atoms [28,29,30].

Thus, in this paper, we attempted to combine the advantages of single-atom Cu and h-BN as catalysts and systematically studied the catalytic mechanism of nitrate to ammonia by single-atom Cu loaded on a monolayer h-BN (SA Cu@h-BN). Firstly, the doping energy was calculated to determine the most stable doping site for a Cu atom on monolayer h-BN, and the band structure and DOS of corresponding structures were analyzed to reveal the influence of single-atom Cu on the reduction ability of monolayer h-BN from the electronic level. Then, the adsorption energies of the NO_3_^−^ ion on monolayer and SA Cu@h-BN were calculated to obtain the most stable adsorption site. Finally, the free energy diagrams for NO_3_^−^ reduction to NH_3_ were obtained to further confirm the rate-determining steps and the corresponding energy barriers. It is expected to provide theoretical guidance for the development of single-atom Cu loaded on h-BN for catalytic reduction of the nitrate to ammonia.

## 2. Results and Discussion

### 2.1. h-BN-Supported Singe-Atom Cu

**Figure 1:**
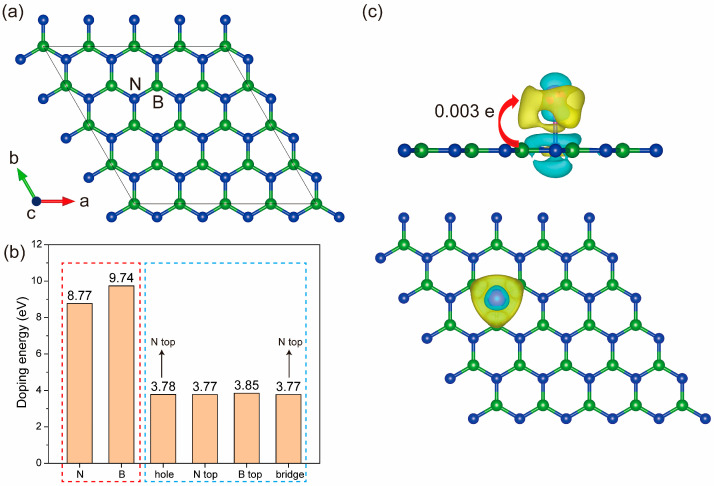
The 4 × 4 supercell of monolayer h-BN, in which the green and blue balls represent B and N atoms, respectively (**a**); the doping energy of Cu in h-BN (**b**); the top view and side view (**c**) of 3D charge difference of singe-atom (SA) Cu-doped h-BN at the N top site (iso-surface value 0.0075 eV/Bohr^3^), in which a Cu atom is represented by a red ball, and the yellow and green regions denote charge accumulation and depletion, respectively.

To study the doping site of singe-atom (SA) Cu on monolayer h-BN, we firstly constructed the (4 × 4) supercell structure of h-BN as shown in Figure 1a. The doping energy was calculated according to the following equation [31]:

(2) $$\Delta E=E_{fin}+E_{t}[B\;or\;N_{2}]-E_{ini}-E_{t}[Cu]$$

where *E_fin_* and *E_ini_* represent the total energies of h-BN after and before Cu doping, respectively; *E_t_* is the total energy of B or Cu elemental substance or isolated N_2_ gas molecule. The calculated results are shown in Figure 1b.

There are two doping methods of SA Cu on monolayer h-BN: substitution and interstitial. Therefore, there exist a total of 6 dopant sites, which are Cu replacing B and N, and Cu inserting at B top, N top, B-N bridge, and hollow sites, respectively. The results in Figure 1b indicate the following: (1) The doping energy at any site is positive, indicating that the doping of SA Cu on monolayer h-BN is an endothermic reaction. Therefore, the lower the doping energy, the easier the doping process is. (2) The doping energy of the interstitial method is significantly lower than that of substitution doping. (3) The doping energy inserted at the hollow, N top, and bridge sites is almost the same (3.77 eV), and it is lower than that at the B top site (3.85 eV). From the optimized coordinates, it can be found that the Cu atom initially located at a hollow or bridge site will move to the N top site after the structural relaxation. This indicates that the N top and B top sites are the stale doping sites, and the N top site is the most optimal doping site, which is because N (3.04) has a stronger Pauling electronegativity than B (2.04), leading to a stronger attraction for the Cu atom.

Furthermore, to study the influence of SA Cu doping on the charge distribution of the monolayer h-BN, the charge difference was calculated and shown in Figure 1c. From Figure 1c, it can be observed that there exists a clear electron exchange between the SA Cu and the monolayer h-BN. The Bader charge calculations indicate that the number of electrons transferred from monolayer h-BN to SA Cu is 0.003 e.

To sum up, due to the stronger electronegativity of the N atom, the Cu atom is preferentially doped at the N top site, resulting in a significant electron rearrangement.

### 2.2. Band Structure and DOS

**Figure 2:**
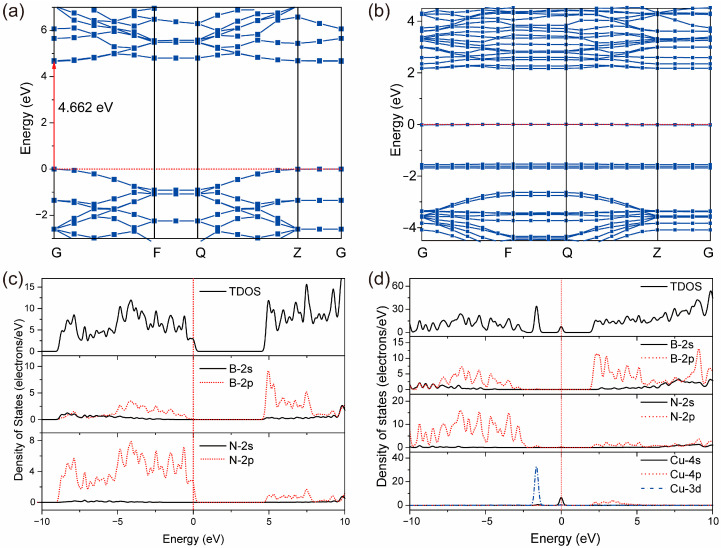
The band structure and density of states (DOS) of monolayer h-BN (**a**,**c**) and SA Cu@h-BN (**b**,**d**). The symbols G, F, Q, and Z in (**a**,**b**) represent the high symmetry K-points over Brillouin zone and the corresponding fractional coordinates are G: (0, 0, 0), F: (0, 0.5, 0), Q: (0, 0.5, 0.5), and Z: (0, 0, 0.5), respectively.

The calculated band structures of monolayer h-BN before and after SA Cu doped at the N top site shown in Figure 2a,b can demonstrate the following results: (1) after Cu doping, an energy band emerges at the Fermi level, leading to the enhancement of the conductivity of monolayer h-BN and the narrowing of the bandgap; (2) the conduction band minimum (CBM) moves toward the Fermi level, thereby improving the reduction capacity of monolayer h-BN. These results are further proved by the density of states (DOS) in Figure 2c,d. In addition, the calculated DOS also shows that (1) the obvious overlap between the 2p orbitals of B and N atoms in the valence band results in the strong covalent interaction between B and N of monolayer h-BN; (2) the new emerging energy bands near the Fermi level and about −1.8 eV originate from the 3d-orbital electrons of the doped Cu atom.

Therefore, the doped Cu atom at the N top site for monolayer h-BN can provide extra 3d-orbital electrons at the Fermi level, which can significantly enhance the conductivity, reduce the bandgap width, and increase the reducibility. This is beneficial for the reduction of NO_3_ to NH_3_.

### 2.3. NO_3_ Adsorption

**Figure 3:**
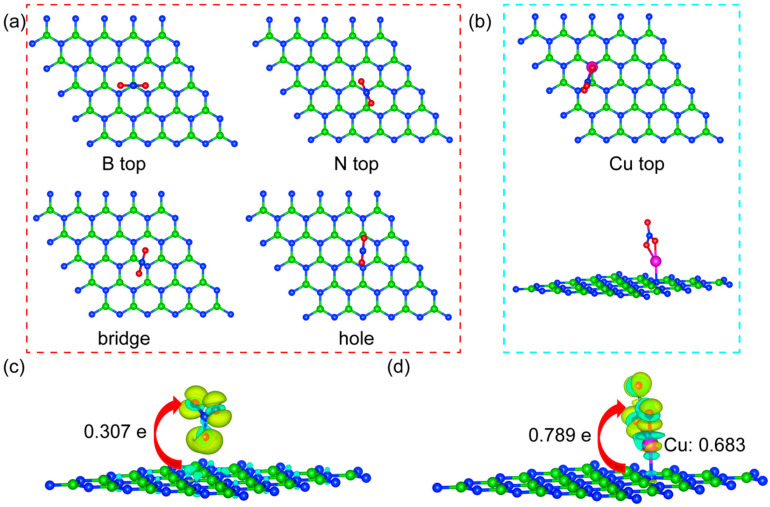
The adsorption site of NO_3_^−^ on monolayer h-BN (**a**) and SA Cu@h-BN (**b**). The 3D charge difference of NO_3_^−^ adsorption on the hollow site of monolayer h-BN (**c**) and Cu top of SA Cu@h-BN (**d**) (iso-surface value 0.0075 eV/Bohr^3^), where the yellow and green regions denote charge accumulation and depletion, respectively. The arrows in (**c**,**d**) indicate the direction of electron transfer.

The adsorption of the NO_3_^−^ ion on the surface is the key step in the reduction to form NH_3_. Figure 3a shows four possible adsorption sites of NO_3_^−^ on monolayer h-BN, including the B top site, the N top site, the bridge site between B and N atoms, and the hollow site located at the center of the hexatomic ring. After SA Cu doped h-BN, we only consider Cu top site. The adsorption energy was calculated by using the following equation [32]:

(3) $$E_{ads}=E_{NO_{3}-ads}-E_{surface}-E_{NO_{3}}$$

where, $E_{NO_{3}-ads}$ and $E_{surface}$ represent the total energies of surface after and before NO_3_^−^ adsorption. $E_{NO_{3}}$ is the total energy of an isolated NO_3_^−^ ion. The calculated adsorption energy *E_ads_*, the initial adsorption site, and the final adsorption site after structural optimization of the NO_3_^−^ ion on monolayer h-BN and SA Cu@h-BN are listed in Table 1.

The calculated results in Table 1 demonstrate that (1) on monolayer h-BN, the optimal site of NO_3_^−^ adsorption on monolayer h-BN is the hollow site (−1.715 eV), the next is the B top site (−1.712 eV), and the worst adsorption site is the N top site (−1.701 eV). The NO_3_ ion adsorption on the bridge site shifts to the B top site after structural relaxation. (2) The adsorption energies of NO_3_^−^ at four sites of monolayer h-BN (about −1.71 eV) are not significantly different, but noticeably weaker than that at the Cu top site of SA Cu@h-BN (−5.554 eV).

To deeply reveal the mechanism of NO_3_^−^ adsorption on monolayer h-BN and SA Cu@h-BN, the charge differences were calculated and shown in Figure 3c,d. Firstly, it can be observed that the NO_3_^−^ ion is adsorbed on the surfaces in a vertical manner, and there is a significant electron rearrangement between the NO_3_^−^ and surfaces. On monolayer h-BN, the NO_3_^−^ ion interacts with the π bond at the center of the h-BN ring through the O atom at the bottom of NO_3_^−^. On SA Cu@h-BN, the two downward-pointing O atoms of NO_3_^−^ form a stronger interaction with the Cu atom. Secondly, the Bader charge calculations indicate that the number of electrons transferred from the surface to the NO_3_^−^ ion increases from 0.307 e to 0.789 e after Cu doping. This is because on SA Cu@h-BN, the Cu atom, as the transition metal, possesses abundant d-orbital electrons, which can be supplied to the NO_3_^−^ ion, thereby generating a stronger interaction.

In summary, on monolayer h-BN, the NO_3_^−^ ion preferentially adsorbs at the hollow site. However, on SA Cu@h-BN, the NO_3_ ion is adsorbed more strongly at the Cu top site due to the abundant d-electron supply from the Cu atom, which is beneficial for the subsequent hydrogen reduction of the NO_3_^−^ ion to NH_3_.

### 2.4. NO_3_ Reduction to NH_3_

**Figure 4:**
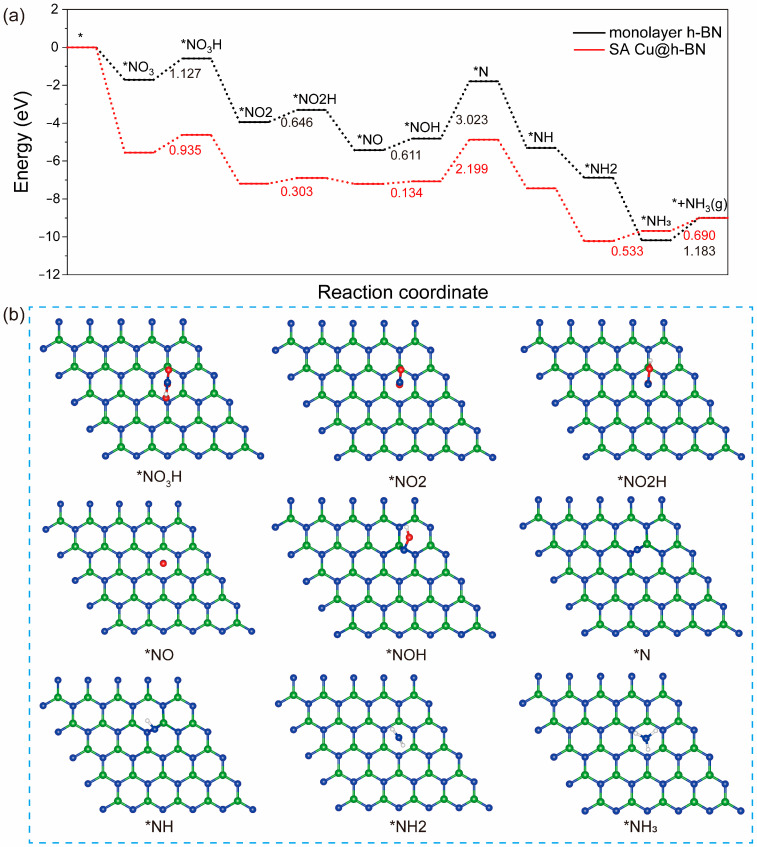
Free energy diagrams for NO_3_^−^ reduction to NH_3_ on monolayer h-BN and SA Cu@h-BN (**a**) and the optimized intermediate adsorption configurations on monolayer h-BN (**b**). All “*” symbols indicate the surface adsorbed structures.

As shown in Figure 4a, the reduction reaction of the NO_3_^−^ ion (NO_3_RR) converted to NH_3_ tends to proceed along the lines of * → *NO_3_ → *NO_3_H → *NO2 → *NO2H → *NO → *NOH → *N → *NH → *NH2 → *NH_3_ → NH_3_. The most stable adsorption structure of *NO_3_H, *NO2, *NO2H, *NO, *NOH, *NH, *NH2, *NH_3_ intermediates on the monolayer h-BN surface are shown in Figure 4b. The relative energies of the optimized intermediate adsorption configurations are listed in Table 2. As shown in Figure 4a, the entire reaction process can be divided into two parts: the reduction of the NO_3_^−^ ion to an N atom, and the hydrogenation of the N atom to form NH_3_.

During the first part of the reduction reaction process, all O atoms in the NO_3_^−^ ion are converted to H_2_O molecules through two steps of hydrogenation reactions in which the bonds between O and N atoms are broken. Among them, the first step of the hydrogenation reactions is an uphill reaction, while for the second step of the hydrogenation reactions, except for the conversion of *NOH to *N which is an uphill reaction, the others can occur spontaneously. On monolayer h-BN, the hydrogenation processes of *NO_3_ → *NO_3_H, *NO2 → *NO2H, *NO → *NOH and *NOH → *N need to overcome the energy barriers of 1.127 eV, 0.646 eV, 0.611 eV, and 3.023 eV, respectively. After single-atom Cu doping, all the corresponding energy barriers are reduced to 0.935 eV, 0.303 eV, 0.134 eV, and 2.199 eV, respectively. Obviously, the hydrogenation reaction from *NOH to *N is the rate-determining step (RDS). The energy barrier of RDS is decreased significantly by the Cu atom supported on monolayer h-BN, which is mainly due to the unique reducing ability of the d orbitals of the Cu atom.

Upon further analyzing the second part of NO_3_RR, it can be observed that three steps of hydrogenation reactions from *N to *NH_3_ on monolayer h-BN are all spontaneous, while the reaction of *NH2 to *NH_3_ on SA Cu@h-BN needs to overcome an energy barrier of 0.533 eV. The final dissociation reaction of NH_3_ is endothermic, requiring 1.183 eV and 0.690 eV of energy on monolayer h-BN and SA Cu@h-BN, respectively. Overall, on the SA Cu@h-BN surface, a total energy barrier of 1.223 eV is required to complete the reaction from *N to NH_3_ gas, which is higher than the 1.183 eV on monolayer h-BN. This is because of the stronger adsorption ability of the Cu atom supported on h-BN.

In summary, the reduction reaction of the NO_3_^−^ ion (NO_3_RR) to NH_3_ can be divided into two parts: the reduction of *NO_3_ to *N and the hydrogenation of *N to NH_3_. The rate-determining steps are the reduction step of *NOH to *N and the dissociation step of NH_3_, respectively. Due to the strong reducing capacity of the d-orbital electrons of the Cu atom, the energy barrier of the RDS can be significantly decreased after the Cu doping of h-BN. However, the dissociation of NH_3_ becomes slightly more difficult. The loading single-atom Cu can improve the catalytic performance of monolayer h-BN, which is consistent with previous experimental results [25].

## 3. Computational Details

In this paper, all first-principles calculations were performed using Density Functional Theory (DFT) [33,34] by utilizing the program package DS-PAW (2023B) of Device Studio (2024A) software [35]. We employed the projector augmented wave (PAW) method [36] and the Perdew–Burke–Ernzerhof (PBE) form of the generalized-gradient approximation (GGA) exchange-correlation energy functional. The structure optimizations were carried out by allowing all atomic positions to vary and relaxing lattice parameters. To accurately calculate the van der Waals force, the DFT-D2 dispersion correction was considered using the Grimme method [37,38]. The calculations would stop once the total energies converged to 10^−6^ eV/atom and the forces on each unconstrained atom were smaller than 0.03 eV/Å. The plane-wave cutoff, *E*_cut_, was chosen to be 400 eV. The k-point mesh of the Brillouin zone automatically was set to 4 × 4 × 1 by using the Gamma Center method.

## 4. Conclusions

In this paper, we have performed a series of first-principles calculations to investigate the catalytic mechanisms of NO_3_^−^ reduction to NH_3_ on monolayer h-BN and SA Cu@h-BN. The following conclusions can be drawn:

1. The Cu atom is preferentially loaded at the N top site of monolayer h-BN, which is because of the stronger electronegativity of the N atom than that of the B atom.
2. The Cu atom supported on monolayer h-BN can enhance electroconductibility, reduce the bandgap width, and increase the reducibility due to its abundant 3d-orbital electrons
3. On monolayer h-BN, the NO_3_^−^ ion preferentially adsorbs at the hollow site, while on SA Cu@h-BN, the NO_3_^−^ ion is adsorbed more strongly at the Cu top site.
4. The rate-determining steps during the reduction of *NO_3_ to *N and the hydrogenation of *N to NH_3_ are the reduction step of *NOH to *N and the dissociation step of NH_3_, respectively. Due to the strong reducing capacity of the d-orbital electrons of the Cu atom, the energy barrier of the RDS can be significantly decreased. Consequently, SA Cu@h-BN exhibits excellent catalytic performance of NO_3_RR.
5. The theoretical calculations in this paper can provide theoretical guidance for the development of h-BN-supported single-atom metal catalysts.

## Figures and Tables

**Table 1:** The adsorption energy *E_ads_*, the initial adsorption site, and the final adsorption site after structural optimization of the NO_3_^−^ ion on monolayer h-BN and SA Cu@h-BN. The “/” represents that there is no change in atomic positions after structural optimization.

System	Initial Adsorption Site	*E_ads_* (eV)	Shifting to
monolayer h-BN	B top	−1.712	/
	N top	−1.701	/
	bridge	−1.711	B top
	hollow	−1.715	/
SA Cu@h-BN	Cu top	−5.554	/

**Table 2:** The relative energies of the optimized intermediate adsorption configurations on monolayer h-BN and SA Cu@h-BN.

System	h-BN	SA Cu@h-BN
*NO_3_	−1.715	−5.554
*NO_3_H	−0.589	−4.618
*NO2	−3.945	−7.195
*NO2H	−3.299	−6.892
*NO	−5.425	−7.207
*NOH	−4.813	−7.073
*N	−1.791	−4.874
*NH	−5.308	−7.437
*NH2	−6.877	−10.223
*NH_3_	−10.184	−9.691
*+NH_3_	−9.001	−9.001

## Data Availability

Research data will be made available on request.
